# WeatherMono: A CNN-Transformer Architecture for Self-Supervised Monocular Depth Estimation in Rainy and Foggy Conditions

**DOI:** 10.3390/s26051705

**Published:** 2026-03-08

**Authors:** Yongsheng Qiu

**Affiliations:** School of Computer Science and Technology, Kashi University, Kashi 844000, China; qys15840212165@163.com

**Keywords:** monocular depth estimation, cnn-transformer, adverse weather conditions, adaptive attention mechanism

## Abstract

In rainy and foggy conditions, the scattering of light and the occlusion effects of atmospheric particles distort the reflected light from object surfaces, leading to inconsistent depth information. As a result, depth estimation models trained under clear weather conditions fail to generalize effectively to adverse weather conditions. To address this challenge, we propose a novel CNN-Transformer architecture, WeatherMono, for self-supervised monocular depth estimation under rainy and foggy weather. Rainy and foggy images often contain large regions of low contrast and blurry features. By combining Convolutional Neural Networks (CNNs) with Transformers, WeatherMono effectively captures both local and global contextual information, thus improving depth estimation accuracy. Specifically, we introduce a Multi-Scale Deformable Convolution (MDC) module and a Global-Local Feature Interaction (GLFI) module. The MDC module extracts detailed local features in rainy and foggy environments, while the GLFI module incorporates an efficient multi-head attention mechanism into the Transformer encoder, enabling more effective capture of both local and global information. This enhances the model’s ability to comprehend image features, strengthens its capability to handle low-contrast and blurry images, and ultimately improves the accuracy of depth estimation in adverse weather conditions. Experiments on WeatherKITTI show WeatherMono achieves AbsRel of 0.097, outperforming WeatherDepth (0.104) and RoboDepth (0.107). On DrivingStereo, it achieves AbsRel of 0.149 (rain) and 0.101 (fog). Extensive qualitative and quantitative experiments demonstrate that WeatherMono significantly outperforms existing methods in terms of both accuracy and robustness under rainy and foggy conditions.

## 1. Introduction

**Figure 1:**
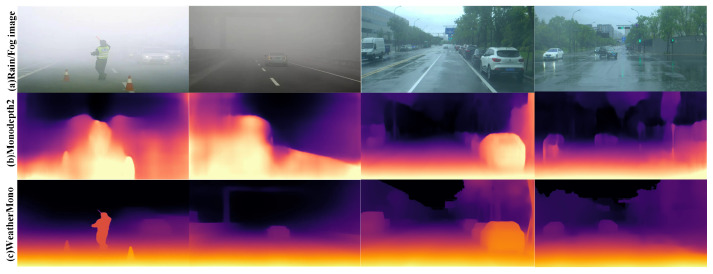
Effectiveness of several classic monocular depth estimation algorithms in rainy and foggy environments. Compared to robust-depth (a state-of-the-art model for robust depth estimation in adverse weather), our WeatherDepth generates more accurate results in rainy and foggy scenes.

Depth estimation is one of the key tasks in computer vision, with widespread applications in image scene understanding, object recognition, 3D reconstruction, and decision-making control, particularly in autonomous driving systems [1,2]. Traditional depth estimation methods typically rely on sensors such as LiDAR or depth cameras to acquire depth information [3]. However, under adverse weather conditions like rain and fog, these sensors are highly susceptible to environmental interference, leading to a significant degradation in depth estimation accuracy. As shown in Figure 1, accurate depth estimation under such challenging weather conditions remains a major challenge [4,5].

In recent years, researchers have proposed using geometric constraints from monocular video for self-supervised depth estimation, achieving accurate and clear results in clear weather conditions. However, in rainy and foggy environments, the scattering and occlusion effects of atmospheric particles significantly degrade the accuracy and robustness of current monocular depth estimation algorithms [6,7]. Some studies have attempted to mitigate the impact of adverse weather on monocular depth estimation by employing image restoration techniques (e.g., rain removal, fog removal) and consistency feature analysis [8]. However, these methods often fail to fully account for the dependency between global and local features in real-world rainy and foggy images, resulting in suboptimal performance in practical applications.

Deep learning, particularly Convolutional Neural Networks (CNNs) and Transformer architectures, has demonstrated exceptional performance in computer vision tasks such as depth estimation. However, CNNs, due to the inherent limitations of their convolutional kernels, can only extract local features within a limited receptive field and are unable to capture long-range global information. To obtain richer global features, CNN-based architectures must increase network depth, which significantly elevates the number of parameters and computational complexity. Transformer-based models, on the other hand, are capable of modeling global context, making them well-suited for monocular depth estimation architectures. However, compared to CNNs, the nonlinear quadratic computational complexity of the Multi-Head Self-Attention (MHSA) module in Transformers results in lower efficiency when processing high-resolution images, failing to meet the requirements for lightweight and fast processing in practical applications. To effectively address the issue of depth inconsistency in rainy and foggy environments, enhance the robustness of monocular depth estimation, and reduce model complexity, this paper proposes a hybrid architecture combining CNN and Transformer. This approach effectively captures both local and global contextual information in rainy and foggy images, further improving depth estimation accuracy.

To effectively address the issue of depth inconsistency in rainy and foggy environments, enhance the robustness of monocular depth estimation, and reduce model complexity, this paper proposes a hybrid CNN-Transformer architecture, WeatherMono, for self-supervised monocular depth estimation under adverse weather conditions. The architecture leverages a Multi-Scale Deformable Convolution (MDC) module and a Global-Local Feature Interaction (GLFI) module. The MDC module extracts detailed local features from rainy and foggy images, while the GLFI module incorporates an efficient multi-head attention mechanism into the Transformer encoder. By introducing a compressed multi-head self-attention mechanism and spatial attention positional encoding, the model maintains computational efficiency while offering flexibility to handle images of varying resolutions. This design allows the model to adaptively adjust attention weights based on the dynamic input features of rainy and foggy images, enhancing its ability to process low-contrast and blurry images. Consequently, this approach improves the accuracy of depth estimation in adverse weather conditions.

The main contributions of this paper can be summarized as follows:

(1) To effectively address the challenges of monocular depth estimation in rainy and foggy environments, we propose a self-supervised monocular depth estimation network (WeatherMono) based on a CNN-Transformer architecture. This network leverages the complementary advantages of CNNs and Transformers to deeply explore the global-local features between rainy/foggy regions and the underlying semantic information of the image, providing an effective solution for monocular depth estimation in adverse weather conditions.

(2) We design a novel Multi-Scale Deformable Convolution (MDC) module and a Global-Local Feature Interaction (GLFI) module. The MDC module extracts detailed local features from rainy and foggy images, while the GLFI module introduces an efficient multi-head attention mechanism into the Transformer encoder, thereby improving the accuracy of monocular depth estimation under rainy and foggy conditions.

(3) Through extensive qualitative and quantitative analysis, as well as ablation experiments, we demonstrate the feasibility and effectiveness of the WeatherMono network for monocular depth estimation in adverse weather conditions such as rain and fog. Furthermore, experiments show that the proposed method achieves a good balance between model complexity and inference speed.

## 2. Related Work

Monocular depth estimation, as a significant research area in computer vision, has made remarkable progress in recent years, largely driven by advancements in deep learning techniques. Monocular depth estimation aims to predict the depth value of each pixel from a single RGB image. Its challenge lies in the lack of stereo matching information, which necessitates the model’s extracting depth information directly from image features. Since monocular depth estimation relies solely on a single image, it is more cost-effective and has broader applicability. Current depth estimation methods can be categorized into three types: monocular depth estimation based on Convolutional Neural Networks (CNNs), monocular depth estimation based on Transformers, and hybrid architectures combining both.

### 2.1. Monocular Depth Estimation Based on Convolutional Neural Networks

Convolutional Neural Networks (CNNs), with their unique network structure, have shown significant advantages in feature extraction for image processing tasks and are widely used in monocular depth estimation. In 2014, Eigen et al. [9] introduced the first deep neural network model for monocular depth estimation, which refines depth prediction through a multi-scale network architecture. Later, Laina et al. [10] proposed a deeper residual network using a new upsampling module and reverse Huber loss to improve training, significantly enhancing depth estimation accuracy. In recent years, many researchers have introduced Fully Convolutional Networks (FCNs), such as the U-Net architecture, to handle high-resolution depth map predictions. Fu et al. [11] employed a multi-scale network, treating depth estimation as an ordered regression task, which not only effectively improved the accuracy of monocular depth estimation but also accelerated model convergence. Since large-scale annotated datasets are not suitable for all scenarios, self-supervised depth estimation methods that do not require ground truth labels have also shown promising results. Zhou et al. [12] proposed a multi-view pose network to estimate the pose between two consecutive frames. To model dynamic scenes, they introduced optical flow estimation [13] and semantic segmentation [14] as additional constraints, improving monocular depth estimation accuracy. Godard et al. [15] proposed the Monodepth2 network, which improves depth estimation accuracy by simply enhancing the loss function without introducing extra constraints. They used minimal reprojection loss to mitigate occlusion issues and employed auto-request loss to filter out moving objects with camera-relative motion. Due to the limited receptive field of CNNs, and to capture long-range dependencies between features, Zhou et al. [16] introduced HRNet for self-supervised monocular depth estimation, incorporating a channel-based attention network to aggregate discriminative features across channel dimensions. Despite ongoing optimization of CNN architectures and increasing network depth, which has improved the accuracy of monocular depth estimation, CNNs still face challenges in local feature extraction and global context modeling.

### 2.2. Monocular Depth Estimation Based on Transformer

Transformer, with its powerful global modeling capabilities, significantly enhances depth estimation accuracy and generalization by capturing global contextual information through the self-attention mechanism. Unlike CNNs, which are limited by local receptive fields, Transformer overcomes the constraint of local perception and better captures long-range dependencies. Traditional CNN-based methods rely on local receptive fields, which limit their ability to model long-range dependencies or understand global scene context. In contrast, Transformer, through self-attention, can capture global contextual information, overcoming the limitations of local perception, and thus demonstrates stronger modeling capabilities in monocular depth estimation tasks.

For example, DepthFormer [17] employs the Swin Transformer as an encoder, efficiently capturing global depth relationships through self-attention, and combines it with a convolutional decoder to generate high-resolution depth maps. By introducing a multi-scale self-attention mechanism, it fuses features from different scales, thereby improving the handling of complex scenes and fine details. In complex scenarios, Transformer’s multi-scale modeling and global context modeling capabilities lead to better edge details and object segmentation in depth maps. However, due to the high computational complexity of Multi-Head Self-Attention (MHSA) in Transformers, the efficiency is lower when processing high-resolution images, which limits its performance in practical applications.

### 2.3. Monocular Depth Estimation Based on Hybrid Architectures

Hybrid architectures for monocular depth estimation combine the strengths of Convolutional Neural Networks (CNNs) and Transformers, leveraging CNN’s powerful local feature extraction ability and Transformer’s global context modeling capacity to provide an efficient and accurate solution for monocular depth estimation tasks. Through careful feature fusion and modular design, hybrid architectures maintain local detail recovery while enhancing the global consistency of depth predictions [18,19]. CNNs excel in extracting local image features, such as edges and textures, making them ideal for capturing fine-scale details. Transformers, on the other hand, can capture long-range dependencies and global context through the self-attention mechanism, making them better suited for handling large-scale structural information. In these hybrid architectures, CNNs are responsible for extracting local features, while Transformers capture global information. By designing specific fusion modules to combine features extracted by CNNs and Transformers, these architectures ensure local detail recovery and improve the global consistency of depth predictions, enabling more accurate depth estimation in complex scenes. Hybrid architectures allow dynamic adjustment of the balance between CNNs and Transformers based on task requirements, thus balancing computational efficiency and prediction performance [20].

Building on the advantages of hybrid network architectures, this paper proposes a novel CNN-Transformer architecture for self-supervised monocular depth estimation under rainy and foggy weather conditions. Rainy and foggy images often contain large areas of low contrast and blurry features. By combining CNNs with Transformers, this architecture effectively captures both local and global contextual information in rainy and foggy images, thereby enhancing depth estimation accuracy. The proposed end-to-end encoder-decoder network architecture utilizes the complementary strengths of CNNs and Transformers to deeply explore the relationship between the image’s rainy/foggy regions and the underlying semantic features, offering an effective solution to the problem of monocular depth estimation in adverse weather conditions.

### 2.4. Depth Estimation in Adverse Environments

In recent years, there has been significant progress in depth estimation tasks under adverse conditions, providing a new research foundation for monocular depth estimation in rainy, foggy, and other harsh environments. Liu et al. [21] improved monocular depth estimation accuracy in nighttime scenes by using Generative Adversarial Networks (GANs) to render night-time environment images and utilizing pseudo-labels estimated during the day to supervise the training of nighttime scenes. Zhao et al. [22] further considered rainy night scenes, employing a dual-branch encoder network structure, where separate encoders were trained on nighttime and daytime image pairs. Consistency constraints were applied to both pixel-level features and depth information to enhance the network’s feature representation. In recent works, Lee et al. [23] proposed a method for depth estimation under adverse weather conditions, combining an image enhancement network to improve depth estimation accuracy. Wang et al. [24] introduced the WeatherDepth network model, which utilizes a self-supervised depth estimation strategy and curriculum contrastive learning to effectively address the performance degradation of monocular depth estimation in adverse weather conditions. Gasperini et al. [6] proposed the md4all model, which generates image samples in complex environments using adversarial neural networks. These generated samples are then input into the model, and corresponding standard losses are computed on the original images to guide supervised training.

To effectively improve the accuracy and robustness of depth estimation in rainy and foggy environments, the monocular depth estimation model proposed in this paper, WeatherMono, employs an end-to-end encoder-decoder network architecture. By leveraging the complementary strengths of CNNs and Transformers, it deeply explores the relationship between rainy/foggy regions and the underlying semantic features of the image, providing an effective solution to the problem of monocular depth estimation in adverse weather conditions.

To provide a clearer overview of the current state-of-the-art, we summarize the key characteristics, limitations, and performances of representative methods in Table 1. As shown in the table, while pure CNN methods (e.g., Monodepth2) suffer from limited global context, Transformer-based methods (e.g., DepthFormer) offer better global modeling but at a higher computational cost. Hybrid CNN-Transformer approaches (e.g., MonoViT, Lite-Mono) attempt to balance these trade-offs, yet they still struggle with the fine-grained local details required for challenging weather scenarios. In contrast, our proposed WeatherMono, with its dedicated MDC and GLFI modules, achieves superior performance (AbsRel 0.097) by jointly enhancing local feature extraction and efficient global context modeling.

## 3. Proposed Method

In this section, we first introduce the overall architecture of the WeatherMono monocular depth estimation network for rainy and foggy environments, and provide a detailed explanation of the encoder-decoder depth estimation network composed of the Multi-Scale Deformable Convolution (MDC) module and the Global-Local Feature Interaction (GLFI) module. Finally, we describe the pose estimation network (PoseNet) and the design of the loss function during the network training process.

### 3.1. Overall Network Architecture

The core contributions of WeatherMono lie in two novel modules: the Multi-Scale Deformable Convolution (MDC) module for enhanced local feature extraction in rainy and foggy conditions, and the Global-Local Feature Interaction (GLFI) module for efficient global context modeling. These modules work synergistically to address the challenges of depth estimation under adverse weather by capturing both fine-grained local details and long-range dependencies.

**Figure 2:**
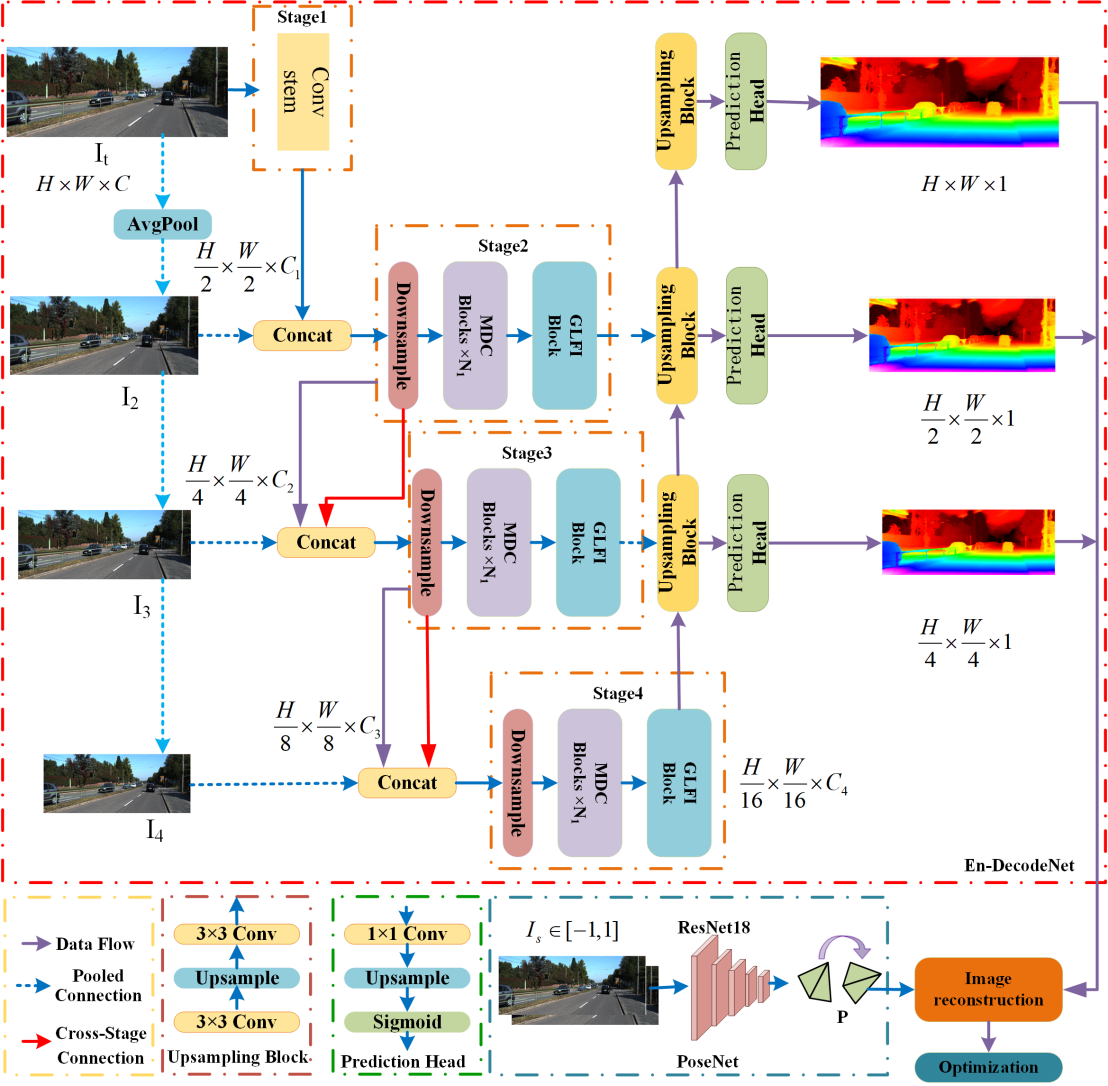
Overall architecture of the monocular depth estimation network (WeatherMono) for rainy and foggy environments. The network mainly consists of the encoder-decoder depth estimation network (En-Decode Net) and the pose estimation network (PoseNet).

The overall architecture of the WeatherMono network is illustrated in Figure 2, consisting of an encoder-decoder depth estimation network (En-Decode Net) and a pose estimation network (PoseNet) [25]. The En-Decode Net is responsible for obtaining depth maps at different scales from monocular images in rainy and foggy environments, while PoseNet estimates the camera motion pose by calculating the variation trends between consecutive frames.

In the En-Decode Net, to enhance the representation of local features without increasing the computational complexity of the network, we incorporate the proposed Multi-Scale Deformable Convolution (MDC) module during the encoding process. This convolution allows the kernel to learn adaptive sampling positions on the input feature map, enabling better handling of occlusions and geometrically complex deformations present in rainy and foggy environments. The MDC module not only expands the receptive field of CNNs but also effectively reduces the introduction of redundant training parameters. Additionally, to improve the model’s ability to express global contextual information and reduce the computational complexity of traditional Transformers, we introduce the Global-Local Feature Interaction (GLFI) module. This module enhances global feature representation while compressing memory through simple depth convolution operations, thus reducing the computational load required by the Multi-Head Self-Attention (MHSA) mechanism within the Transformer block.

The overall workflow of WeatherMono can be summarized as follows: First, the input image is passed through the encoder, where the MDC module extracts multi-scale local features at each stage, and the GLFI module captures global contextual information. The decoder then upsamples the encoded features using bilinear upsampling and combines them with encoder features from different stages via convolutional layers to produce depth maps at multiple scales (full, 1/2, 1/4 resolution). Finally, the pose estimation network (PoseNet) computes the camera motion between consecutive frames, and the depth estimation network is trained using a combination of image reconstruction loss and perceptual smoothness loss in a self-supervised manner. Detailed descriptions of the MDC and GLFI modules are provided in Section 3.2.

### 3.2. Encoder-Decoder Depth Estimation Network

The encoder part of the WeatherMono network is shown in Figure 2. It decomposes the input image in a rainy or foggy environment into four stages for multi-scale feature aggregation. First, the input image of size H×W×3 is passed through a 3×3 convolution layer for the first stage downsampling. Then, two 3×3 convolutions with a stride of 1 are applied to extract local features, resulting in a feature map of size $\frac{H}{2}\times \frac{W}{2}\times C_{1}$. In the second stage, to reduce spatial information loss caused by feature size reduction, the features obtained from average pooling are concatenated with the output from the first stage. A downsampling operation with a stride of 2 is then applied, yielding a feature map of size $\frac{H}{4}\times \frac{W}{4}\times C_{2}$. This is followed by the proposed Multi-Scale Deformable Convolution (MDC) module and the Adaptive Global-Local Feature Interaction (GLFI) module, which further extract both local and global features. The input to the downsampling layer in the third stage is similarly obtained by concatenating the output from the previous stage and the average pooled features. The feature extraction process in the third and fourth stages is similar to that of the second stage, and the output feature dimensions are $\frac{H}{8}\times \frac{W}{8}\times C_{3}$ and $\frac{H}{16}\times \frac{W}{16}\times C_{4}$.

The MDC module extracts multi-scale local features using deformable convolutions. Unlike the typical use of deformable convolutions only in the last layer of the network, in this work, several consecutive deformable convolutions are inserted in each layer from the second to the fourth stages of the encoder. This allows the model to dynamically learn appropriate local features from different scale features and adaptively adjust sampling offsets and modulation scalars based on the input data. Similar to Vision Transformers (ViTs), this reduces the over-inductive bias inherent in conventional convolutions.

For any point on the input feature map, a traditional convolution operation can be expressed as:

(1) $$y\left(p_{0}\right)=\sum_{p_{n}\in R}w\left(p_{n}\right)\times x\left(p_{0}+p_{n}\right)$$

(2) R={(−1,−1),(−1,0),…,(0,0),…,(1,0),(1,1)}

where $p_{n}$ represents the offset of each point in the convolution kernel relative to the center point, which can be expressed by Equation (2) (taking a 3 × 3 convolution kernel as an example). $w(p_{n})$ denotes the weight at the corresponding position of the convolution kernel, and $x(p_{0}+p_{n})$ represents the element value at the location $p_{0}+p_{n}$ on the input feature map. The principle of deformable convolution is based on a network learning the offset, which causes the convolution kernel to shift its sampling points on the input feature map, focusing on regions or targets of interest. Deformable convolution introduces an offset for each point based on Equation (1), where the offset is learned by the network and is generated through another convolution operation on the input feature map:

(3) $$y\left(p_{0}\right)=\sum_{p_{n}\in R}w\left(p_{n}\right)*x(p_{0}+p_{n}+\Delta p_{n})$$

where $\Delta p_{n}$ represents the offset. Since the position after adding the offset is typically a decimal and does not correspond to actual pixel points on the input feature map, interpolation is required to obtain the pixel values at the offset position. Bilinear interpolation is commonly used for this purpose, and its calculation formula is as follows:

(4) $$\begin{array}{cc} x(p) & =\sum_{q}G\left(q,p\right)\cdot x\left(q\right)\\ \; & =\sum_{q}g\left(q_{x},p_{x}\right)\cdot g\left(q_{y},p_{y}\right)\cdot x\left(q\right)\\ \; & =\sum_{q}\max(0,1-|q_{x}-p_{x}\left|)\cdot \max(0,1-\right|q_{y}-p_{y}\left|\right)\cdot x\left(q\right) \end{array}$$

where

$$\max(0,1-|q_{y}-p_{y}|)$$

ensures that the distance between the interpolation point and the neighboring points does not exceed one pixel.

**Figure 3:**
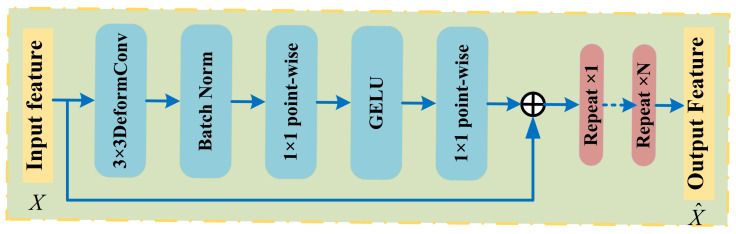
Structure Diagram of the MDC Module.

By using deformable convolution, the network can maintain a fixed size for the output feature map while more flexibly obtaining a larger receptive field. For example, if the input feature *X* has dimensions H×W×C , the output $\hat{X}$ of the MDC module is as follows:

(5) $$\hat{X}=X+Linear_{G}\left(Linear\left(BN\left(DeformConv\left(X\right)\right)\right)\right)$$

where $Linear_{G}$ refers to a pointwise convolution operation using the GELU activation function. BN denotes the batch normalization layer, and DeformConv(·) represents a 3 × 3 deformable convolution. The MDC network architecture is shown in Figure 3.

**Figure 4:**
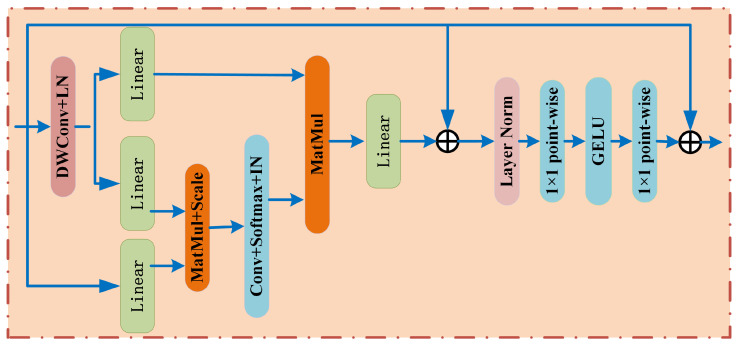
Structure Diagram of the GLFI Module.

The Global-Local Feature Interaction module (GLFI) primarily utilizes an efficient multi-scale Transformer architecture to capture the global contextual information of the input image features. The network architecture diagram is shown in Figure 4. To effectively address the computational and memory issues of the MSA (Multi-Scale Attention) module, this module employs simple depthwise convolutions for memory compression and performs projection interactions across attention head dimensions, effectively reducing the computation and memory requirements of MSA in the Transformer block. The traditional Transformer is mainly composed of two modules: MSA and FFN. For the input feature $x\in^{\;n\times d_{m}}$ , where *n* denotes the spatial dimensions and $d_{m}$ denotes the channel dimension, the output can be expressed as:

(6) $$y=x^{\prime }+\mathrm{FFN}\left(\mathrm{LN}\left(x^{\prime }\right)\right),\mathrm{and}\;x^{\prime }=x+\mathrm{MSA}\left(\mathrm{LN}\left(x\right)\right)$$

where self-attention mechanism is represented by three sets of linear projections mapping the query (Q), key (K), and value (V) as follows:

(7) $$\mathrm{SA}\left(Q,K,V\right)=\mathrm{Softmax}\left(\frac{QK^{T}}{\sqrt{d_{k}}}\right)V$$

As can be seen from the above equation, the computational complexity of MSA increases quadratically with respect to both the spatial and channel dimensions, leading to a significant rise in the training complexity and inference load of the entire network model. To address this issue in traditional Transformers, this paper compresses the memory by first expanding the 2D input $x\in^{\;n\times d_{m}}$ along the spatial dimensions into a 3D form $\hat{x}\in^{\;d_{m}\times h\times w}$ , then passing it through a depthwise convolution to reduce the spatial domain dimensions [1,3]. The resulting feature is in 2D form and is fed into the next two projection sets to obtain K and V. Subsequently, the attention feature output EMSA is computed using Equations (8).

(8) $$\mathrm{EMSA}\left(Q,fK,V\right)=\mathrm{IN}(\mathrm{Softmax}\left(\mathrm{Conv}\left(\frac{QK^{T}}{\sqrt{d_{k}}}\right)\right))V$$

where Conv refers to a 1 × 1 convolution, and IN denotes instance normalization. Finally, the output values from each attention head are concatenated and linearly projected to form the final feature output.

The decoder part of the WeatherMono network is shown on the right side of Figure 2. Unlike approaches that use complex upsampling methods or additional attention modules, we design a simple yet efficient decoder. This decoder expands the spatial dimensions using bilinear upsampling and connects features from three different stages of the encoder via convolutional layers. Specifically, after each upsampling module, a prediction head follows to output the predicted depth map at resolutions of 1, 1/2, and 1/4. This design not only simplifies the network architecture but also significantly improves computational efficiency.

The decoder employs bilinear upsampling, which is a low-cost upsampling method compared to more complex techniques such as deconvolution or pixel shuffle. Bilinear upsampling allows for faster spatial dimension expansion while preserving image luminance information well. Studies have shown that bilinear upsampling excels in maintaining image details and edge information, which is crucial for improving the accuracy of depth estimation tasks.

Furthermore, convolutional layers are used to connect features from different stages of the encoder, enabling the integration of diverse semantic information and spatial resolution extracted at various encoder stages. By merging these features via convolution, the network can leverage information from multiple stages, forming a richer feature representation. This multi-scale feature fusion approach enhances the robustness and accuracy of depth estimation, particularly in complex rain and fog environments, where it better captures and expresses depth information.

Additionally, each upsampling block is followed by a prediction head that outputs depth maps at different resolutions. This multi-resolution output design provides more diverse information sources for subsequent depth fusion. In practical applications, depth maps at different resolutions can complement each other, improving the overall depth estimation’s precision and reliability. Through this efficient and simple depth decoder design, WeatherMono not only reduces computational complexity but also enhances depth estimation performance, especially in challenging rain and fog environments, where it can more accurately estimate depth information for complex scene imagery.

### 3.3. Pose Estimation Network (PoseNet)

In monocular depth estimation tasks, the PoseNet pose estimation network plays a crucial role [12,15]. By predicting the relative pose using PoseNet, depth information from multiple monocular frames can be integrated with the scene’s geometric consistency, thereby improving the accuracy of depth estimation. PoseNet is primarily used to estimate the camera pose at different time points, including both translation and rotation, helping the depth estimation network better understand and reconstruct the 3D structure of the scene.

PoseNet typically consists of a Convolutional Neural Network (CNN). In this work, we use a pre-trained ResNet18 as the encoder for the pose estimation network. The network takes paired images (consecutive frames) as input and learns the variation information between these images through feature extraction layers, as shown in the lower part of Figure 2. The output of PoseNet is the six degrees of freedom (6-DOF) camera pose, consisting of three translation parameters and three rotation parameters.

To train the PoseNet network, two types of losses are used: optical flow consistency loss and pose regression loss. The optical flow consistency loss minimizes the reprojection error by comparing the predicted depth map and the estimated camera pose, while the pose regression loss directly minimizes the error between the predicted and ground-truth poses. The optical flow consistency loss aligns the pixels between consecutive frames using the predicted depth map $D_{t}$ and the relative camera pose $R_{t}$, ensuring geometric consistency by minimizing the photometric error between the current frame image $I_{t}$ and the reconstructed image ${\hat{I}}_{t}$, as follows:

(9) $$L_{o}=\sum_{n\in L}\|I_{t}\left(p\right)-{\hat{I}}_{t}\left(p\right){\|}_{1}+\lambda \cdot \mathrm{SSIM}\left(I_{t},{\hat{I}}_{t}\right)$$

where ${\hat{I}}_{t}\left(p\right)$ represents the pixels of the reference frame reconstructed using the depth map and camera pose. SSIM [26] is the Structural Similarity Index, which is used to preserve the structural information of the image. λ is a hyperparameter that balances the photometric error and the structural similarity error.

The pose regression loss directly calculates the difference between the predicted camera pose and the ground truth pose. This loss typically consists of two components:

Rotation Loss: The rotation loss measures the difference between the predicted rotation $\hat{R}$ and the ground truth rotation *R*. This is commonly expressed using either quaternions or rotation matrices.

(10) $$L_{r}=\|\hat{R}-R{\|}_{F}$$

where $\|\cdot {\|}_{F}$ denotes the Frobenius norm.

Translation Loss: The translation loss computes the Euclidean distance between the predicted translation vector and the ground truth translation vector:

(11) $$L_{t}=\|\hat{t}-t{\|}_{2}$$

The total pose regression loss can be expressed as:

(12) $$L_{p}=\alpha \cdot L_{r}+\beta \cdot L_{t}$$

where α and β are hyperparameters that control the weights of the rotation and translation losses.

By combining the flow consistency loss and the pose regression loss, the network can better learn the pose changes between images. During training, PoseNet and the depth estimation network are typically jointly trained. The depth estimation network is responsible for generating the depth maps, while PoseNet estimates the camera pose. Through joint training, the consistency between the two networks in the feature space is ensured, which in turn improves the overall accuracy of the depth estimation network.

### 3.4. Monocular Depth Estimation Loss Function

Due to the limited availability of ground truth depth annotations for real images in rainy and foggy environments, this paper adopts a self-supervised training approach, transforming the monocular depth estimation task in such conditions into an image reconstruction task. Consequently, the training constraints of the network model include the image reconstruction loss $L_{r}$ between the input image $I_{t}$ and the synthetic target image ${\hat{I}}_{t}$, as well as the perceptual smoothness loss $L_{smooth}$ of the predicted depth map under rainy and foggy conditions.

Image Reconstruction Loss is used to measure the difference between the reconstructed image and the target image. In the self-supervised monocular depth estimation task under rainy and foggy conditions proposed in this paper, the image reconstruction loss is typically used to optimize the model by evaluating the difference between the reprojected generated image and the original image:

(13) $$L_{r}=\sum_{i.j}\left|I_{t}-{\hat{I}}_{t}\right|$$

where $I_{t}$ is the input target image, and ${\hat{I}}_{t}$ is the image generated after reprojection using the depth and pose estimation. The photometric reprojection loss is used to measure the photometric difference between the original image and the synthetic image. This loss combines image reconstruction and depth estimation by minimizing the image reconstruction error, thereby improving the accuracy of depth estimation. Given the target image $I_{t}$ and the source image $I_{s}$, the depth map $D_{t}$ predicted by the depth estimation network, and the relative pose $T_{t\to s}$ predicted by the pose estimation network, the synthetic image ${\hat{I}}_{t}$ can be generated through reprojection. The photometric reprojection loss can be defined as:

(14) $$L_{p}\left({\hat{I}}_{t},I_{t}\right)=L_{p}\left(F\left(I_{s},T_{t\to s},D_{t},K\right),I_{t}\right)$$

where *K* is the camera-specific parameter.

The perceptual loss $L_{p}$ is calculated as the sum of the structural similarity (SSIM) between $I_{t}$ and ${\hat{I}}_{t}$, and the L1 loss:

(15) $$L_{p}\left({\hat{I}}_{t},I_{t}\right)=\alpha \frac{1-SSIM({\hat{I}}_{t},I_{t})}{2}+\left(1-\alpha \right)\left\|{\hat{I}}_{t}-I_{t}\right\|$$

where the parameter α is empirically set to 0.85 [15]. In addition, to suppress the effects of rain and fog occlusions in the source image, the minimum photometric loss is calculated as:

(16) $$L_{p}\left(I_{s},I_{t}\right)=\min_{I_{s}\in \left[-1,1\right]}L_{p}\left({\hat{I}}_{t},I_{t}\right)$$

Furthermore, to exclude the misaligned pixels between adjacent frames, a binary mask layer is introduced:

(17) $$M=\min_{I_{s}\in \left[-1,1\right]}L_{p}\left(I_{s},I_{t}\right)>\min_{I_{s}\in \left[-1,1\right]}L_{p}\left({\hat{I}}_{t},I_{t}\right)$$

Thus, the image reconstruction loss can be defined as:

(18) $$L_{r}\left({\hat{I}}_{t},I_{t}\right)=M\cdot L_{p}\left(I_{s},I_{t}\right)$$

The Edge-aware Smoothness Loss in monocular depth estimation is used to ensure that the predicted depth map remains smooth in flat regions, while allowing depth variations at image edges [16]. This helps preserve details in the edge regions, reduces noise in the depth map, and enhances the quality of the depth map by incorporating image gradient information. The calculation process is as follows:

(19) $$L_{smooth}=\sum_{i,j}\left(\left|\partial_{x}D_{i,j}\right|e^{-\left|\partial_{x}I_{i,j}\right|}+\left|\partial_{y}D_{i,j}\right|e^{-\left|\partial_{y}I_{i,j}\right|}\right)$$

where $\partial_{x}$ and $\partial_{y}$ represent the gradients in the *x* and *y* directions, respectively, and $D_{i,j}$ represents the depth value at pixel position (i,j). Therefore, the total loss function for the WeatherMono monocular depth estimation network in foggy environments can be expressed as:

(20) $$L=\frac{1}{3}\sum_{s\in \{1,\frac{1}{2},\frac{1}{4}\}}\left(L_{r}+\lambda L_{smooth}\right)+L_{p}$$

where *s* denotes the three different scales predicted by the decoder network, and the hyperparameter λ is empirically set to $e^{-3}$.

## 4. Experiments and Analysis

In this section, we first introduce the basic experimental setup, dataset selection, and both subjective and objective evaluation methods for depth estimation. Then, we conduct a comprehensive comparison with current classic monocular depth estimation algorithms through multiple qualitative and quantitative analyses to verify the superiority of the proposed monocular depth estimation method under foggy conditions. Finally, we perform several ablation studies to validate the effectiveness and rationality of the key components in the proposed network model. Through these extensive experimental analyses, we demonstrate that the proposed monocular depth estimation algorithm not only effectively mitigates the inconsistencies in depth information in foggy environments but also alleviates the impact of fog occlusion on monocular depth estimation while enhancing the robustness of the model in challenging conditions.

### 4.1. Implementation Details

The monocular depth estimation network is designed based on the PyTorch framework (version 1.10) and trained on a single NVIDIA GeForce GTX 2080Ti GPU. We employ the Adam optimizer with $\beta_{1}=0.9$, $\beta_{2}=0.9$, and a weight decay of $1\times 10^{-2}$. The initial learning rate is set to $1\times 10^{-4}$ for the depth estimation network and to $5\times 10^{-4}$ PoseNet. The batch size is set to 2 due to GPU memory constraints, and the model is trained for a total of 50 epochs. The learning rate is adjusted using a cosine decay schedule without restarts. To prevent overfitting of the MDC and GLFI modules, we employ Drop-path regularization with a rate of 0.1. During training, data augmentation techniques such as random horizontal flipping, brightness adjustment (±0.2), saturation adjustment (±0.2), and contrast adjustment (±0.2) are applied to improve the robustness of the model.

### 4.2. Datasets and Evaluation Metrics

In this study, the depth estimation dataset for the foggy environment is based on the WeatherKITTI dataset proposed by Wang et al. [24], which includes rain and fog weather conditions. The rain and fog images are simulated using an atmospheric scattering model. A total of 14,000 foggy images with a size of 1280 × 384 are selected from the dataset. This dataset encompasses a wide range of weather severities, from light mist to dense fog and light drizzle to heavy rain, ensuring that the evaluation results in Table 1 reflect model performance across diverse weather intensities. Additionally, to enhance the robustness of the model in real-world environments, 1000 real rain and fog traffic images from the DrivingStereo [27] dataset are incorporated.

To evaluate the overall performance of monocular depth estimation in foggy environments more accurately and objectively, several evaluation metrics are employed: Absolute Relative Error (AbsRel), Root Mean Square Error (RMSE), Root Mean Square Error of Logarithmic Depth (RMSE log), Squared Relative Error (SqRel), and Threshold Accuracy $\left(a_{1},a_{2},a_{3}\right)$ [9]. These metrics evaluate the performance of monocular depth estimation models from various angles, providing a comprehensive reflection of model accuracy and allowing for detailed analysis in specific scenarios.

The Absolute Relative Error (AbsRel) is used to measure the relative error between predicted depth and ground truth depth. It compares the ratio of predicted depth to true depth for each pixel and then averages the results to provide a global error evaluation. This metric helps assess the model’s performance in different scenarios by reducing the influence of large errors at varying distances. The formula for AbsRel is as follows:

(21) $$\mathrm{AbsRel}=\frac{1}{\left|N\right|}\sum_{i\in N}\frac{\left|{\hat{d}}_{i}-d_{i}\right|}{d_{i}}$$

where *N* is the number of pixels in the image, ${\hat{d}}_{i}$ is the true depth at pixel *i*, and $d_{i}$ is the predicted depth at pixel *i*.

Root Mean Square Error (RMSE) is a traditional metric used to measure regression error. It is calculated by summing the squared differences between predicted and true depth, averaging them, and then taking the square root. RMSE gives equal weight to all prediction errors, regardless of whether they are large or small. The formula for RMSE is:

(22) $$\mathrm{RMSE}=\sqrt{\frac{1}{N}\sum_{N}^{i=1}{\left({\hat{d}}_{i}-d_{i}\right)}^{2}}$$

Root Mean Square Error of Logarithmic Depth (RMSE log): This metric is designed to reduce the impact of large errors at greater distances by applying a logarithmic transformation to the depth difference. The logarithmic transformation provides a more reasonable error measurement with respect to depth. The formula for RMSE log is:

(23) $$RMSE_{\log10}=\frac{1}{N}\sum_{i\in N}\left|\log_{10}{\hat{d}}_{i}-\log_{10}d_{i}\right|$$

Squared Relative Error (SqRel) is calculated by taking the squared difference between the predicted and true depth, divided by the true depth, and then averaging the results. This metric is particularly useful for penalizing large depth errors, such as discontinuities in closer objects, offering a more granular assessment of model performance. The formula for SqRel is:

(24) $$\mathrm{SqRel}=\frac{1}{N}\sum \frac{{\left|d_{i}-{\hat{d}}_{i}\right|}^{2}}{d_{i}}$$

Threshold Accuracy $\left(a_{1},a_{2},a_{3}\right)$ measures the percentage of predicted depths that are within a certain threshold of the true depths. The threshold values $\left(a_{1},a_{2},a_{3}\right)$ are typically set to $\left(1.25,1.25^{2},1.25^{3}\right)$. These thresholds determine whether the predicted depth is within a reasonable range of the true depth. The accuracy at each threshold is calculated as follows:

(25) $$\max\left(\frac{{\hat{d}}_{i}}{d_{i}},\frac{d_{i}}{{\hat{d}}_{i}}\right)=a_{i}<1.25^{i}$$

### 4.3. Comparison with State-of-the-Art Methods

To further validate the accuracy and robustness of the WeatherMono depth estimation network in foggy and rainy weather environments, a comprehensive qualitative and quantitative analysis is performed by comparing it with several representative monocular depth estimation algorithms and weather-specific algorithms. These include Monodepth2 [15], Lite-Mono [1], MonoFormer [19], DepthFormer [17], PlaneDepth [28], DepthHints [29], HR-Depth [30], CADepth-Net [31], Manydepth [32], DynaDepth [33], DiFFNet [16], DPT [18], FSRE-Depth [14], ADDS-Depth [21], MonoVit [20], RoboDepth [34], Md4all [6], WeatherDepth [24].

Since the training samples for different methods vary, for those methods that provide pre-trained models, the best pre-trained model is used for objective evaluation. For methods without pre-trained models, the publicly available code is used, and models are retrained with parameter adjustments to ensure a fair comparison. All models are trained and evaluated according to the original settings. During the testing phase, the WeatherMono network is evaluated on multiple foggy image datasets to perform both qualitative and quantitative analysis.

#### 4.3.1. Quantitative Analysis of Monocular Depth Estimation in Rainy and Foggy Conditions

First, we compared the performance of several advanced monocular depth estimation algorithms on a rainy and foggy test dataset. The experimental results are shown in Table 2. This test dataset includes images with varying degrees of rain and fog effects, ensuring a comprehensive and objective reflection of the results. The models are evaluated using various objective metrics, such as AbsRel, RMSE, RMSE log, SqRel, and Threshold Accuracy $\left(a_{1},a_{2},a_{3}\right)$, providing an accurate and objective evaluation of the overall performance of different monocular depth estimation algorithms.

From Table 2, it can be seen that WeatherMono performs excellently in all objective evaluation metrics, particularly in RMSE and SqRel. Compared to WeatherDepth, WeatherMono improves by 3%in RMSE and 5% in SqRel. In contrast, algorithms such as DepthFormer, MonoVit, and MonoFormer, although performing well in clear weather conditions, show considerable fluctuations in depth estimation accuracy in complex and dynamic rain and fog scenes.

For CNN-based monocular depth estimation algorithms, such as the classic Monodepth2, depth estimation accuracy significantly decreases in foggy images. This is due to the inability to effectively filter out the interference caused by fog in the images. Despite the optimization of CNN architectures and increasing network depth, the issue of insufficient global feature extraction and long-term contextual dependencies remains. On the other hand, Transformer-based depth estimation methods are more adaptable, capable of handling depth estimation tasks in foggy environments more effectively, outperforming traditional CNN methods in all objective evaluation metrics. For instance, DepthFormer introduces multi-scale self-attention mechanisms to fuse features from different scales, allowing it to better capture details in foggy environments. However, it suffers from high computational complexity and slower inference speed.

Hybrid architectures combining CNNs and Transformers, such as MonoViT, leverage the strengths of CNNs in local detail perception and Transformers in global long-range feature extraction. These hybrid approaches improve depth estimation accuracy in foggy environments compared to CNN-only or Transformer-only architectures, but they come with increased model complexity and slower inference times.

Compared to the above classic monocular depth estimation algorithms, WeatherMono shows consistent improvement across several objective evaluation metrics in foggy environments. This can be attributed to two main factors:

(1) WeatherMono fully utilizes the complementary advantages of CNNs and Transformers, extracting global-local features from both fog regions and the underlying semantics of the image.

(2) The Multi-scale Deformable Convolution (MDC) module effectively captures fine local details in foggy images, while the Global-Local Feature Interaction (GLFI) module introduces an efficient multi-head attention mechanism in the Transformer encoder to enhance depth estimation accuracy in foggy environments.

To further validate the performance of the WeatherMono network in real-world foggy environments, real rain and fog images from the DrivingStereo dataset are tested. The results are shown in Table 3. It is important to note that all models compared in Table 3 were evaluated in a zero-shot manner, i.e., they were trained on clear-weather or synthetic weather datasets and directly tested on real-world rainy and foggy images from DrivingStereo without any fine-tuning. This evaluation protocol rigorously assesses the generalization capability of each method to unseen real-world adverse conditions. From Table 3, it can be observed that WeatherMono outperforms other existing weather-specific monocular depth estimation algorithms on the DrivingStereo test dataset. This demonstrates its adaptability in dynamic rain and fog scenarios and its ability to effectively mitigate the interference caused by raindrops and fog in depth estimation.

#### 4.3.2. QualitativeAnalysis of Monocular Depth Estimation in Rainy and Foggy Conditions

**Figure 5:**
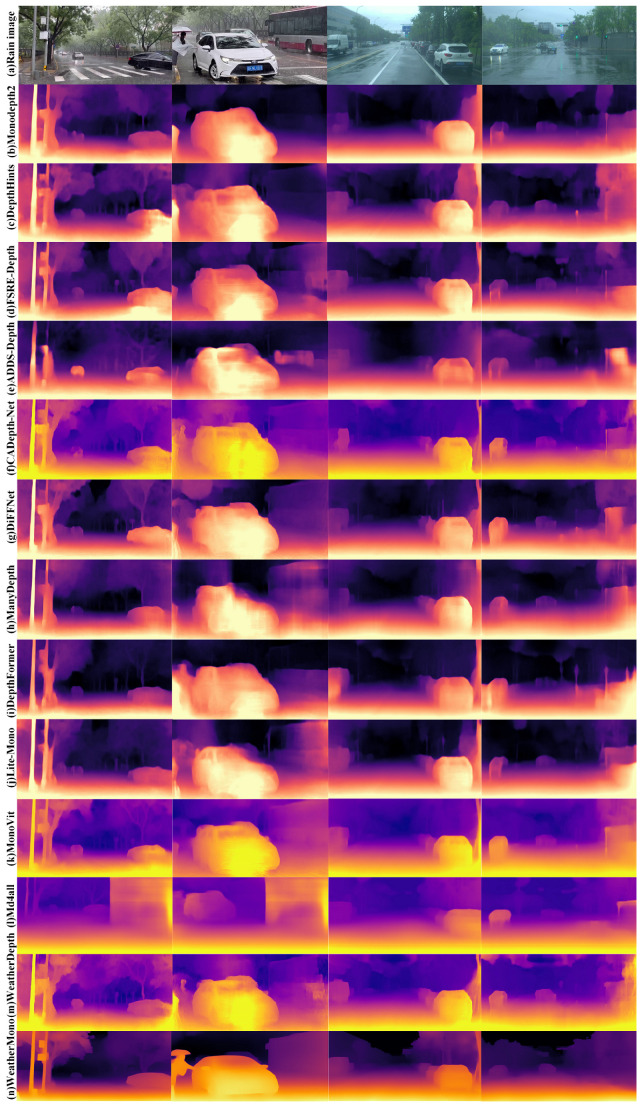
Visual Comparison of Depth Estimation Algorithms in Rainy Environments.

**Figure 6:**
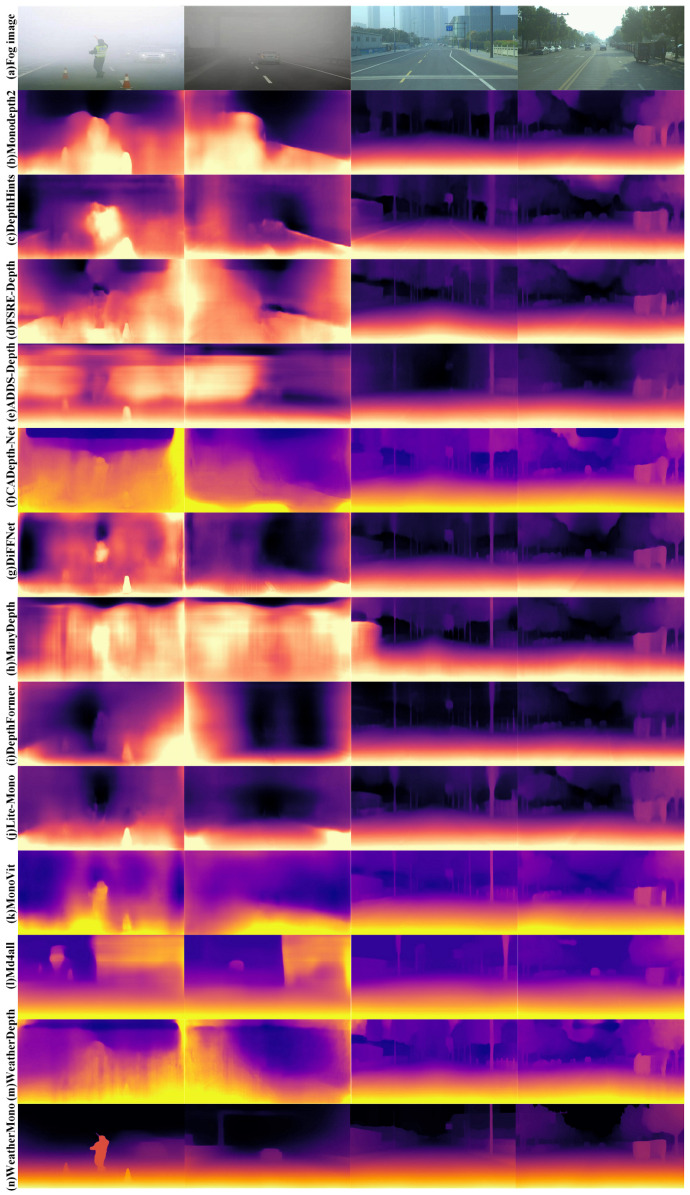
Visual Visual Comparison of Depth Estimation Algorithms in Foggy Environments.

To provide a more intuitive demonstration of the effectiveness of the WeatherMono network in monocular depth estimation under rain and fog conditions, this section presents a qualitative comparison of the WeatherMono model with other classic depth estimation models. The visual comparison results on real rainy and foggy images are shown in Figure 5 and Figure 6.

From the visual results, it is evident that convolutional neural network (CNN)-based monocular depth estimation models struggle with accurately estimating the thickness of fog and the density of rain streaks in images under rainy and foggy conditions. This leads to the inability to effectively suppress the noise interference from fog and raindrops, resulting in errors in depth estimation in certain areas. For example, in rainy conditions, CNN-based models tend to misestimate the depth of regions where visibility is impaired due to heavy rain or fog.

In contrast, Transformer-based methods show stronger adaptability to depth estimation tasks in rainy and foggy environments. However, errors still occur, especially in regions where the fog and raindrops are highly variable. DepthFormer, for instance, does improve on handling fog-induced noise, but the non-uniform nature of fog and rain still causes inaccuracies in depth predictions, especially for distant objects and regions with low contrast.

Hybrid architectures that combine CNNs and Transformers, such as MonoViT and Lite-Mono, show improvements in eliminating some of the fog’s interference. However, these methods often suffer from the degradation of edge and contour details in the images. Specifically, these models tend to omit depth information for small objects, which can lead to inaccurate depth estimations for such objects. For example, both MonoViT and Lite-Mono miss the depth of smaller objects, particularly in foggy areas where visibility is reduced.

From the qualitative comparison results, it is clear that the WeatherMono network exhibits distinct advantages in depth estimation under foggy and rainy conditions. The CNN-Transformer hybrid architecture of WeatherMono is highly effective in reducing the interference from fog and rain droplets while simultaneously improving the consistency of depth information in rainy environments. By effectively capturing both local and global contextual information in the image, WeatherMono significantly enhances the network’s understanding of image features. This allows the model to better handle low-contrast and blurred images, which are common in foggy and rainy environments, thus improving the accuracy of depth estimation under these challenging conditions.

Notably, as shown in Figure 5 and Figure 6, WeatherMono demonstrates robust performance across varying weather intensities. In regions with light fog, the model preserves fine structural details, while in areas with dense fog, it maintains coherent depth structures despite significant visibility degradation. Similarly, under heavy rain, the model effectively handles the occlusion effects of rain streaks, producing smoother and more accurate depth maps compared to baseline methods.

It is worth noting that while the quantitative improvements in Table 1 (e.g., AbsRel: 0.104 vs. 0.097) appear modest numerically, the visual improvements in Figure 5 and Figure 6 are more pronounced. This can be attributed to two factors. First, the evaluation metrics such as AbsRel and RMSE provide a global average over all pixels, which may dilute localized improvements in challenging regions such as distant objects, edges, and areas with heavy fog or rain. Second, WeatherMono’s MDC and GLFI modules are specifically designed to enhance feature extraction in precisely these challenging regions—adaptively sampling local details with deformable convolutions and capturing long-range contextual dependencies with efficient attention. As a result, the visual quality in perceptually important areas (e.g., object boundaries, distant scenery) improves substantially, even if the global pixel-wise error metrics show only incremental gains.

The visual results highlight how WeatherMono can preserve important depth information even in regions with heavy fog and rain, maintaining the structural integrity of the depth maps and offering more accurate predictions compared to other state-of-the-art methods. This makes WeatherMono particularly valuable for applications that require reliable depth estimation in adverse weather conditions, such as autonomous driving and robotics.

### 4.4. Ablation Study on Model Architectures

In this section, we conduct a series of ablation studies to demonstrate the effectiveness of each module in the proposed WeatherMono monocular depth estimation model. By removing or replacing individual components, we aim to validate the contribution of key modules, including the Multi-Scale Deformable Convolution (MDC), Global-Local Feature Interaction (GLFI) module, pooling connections, and cross-stage connections, in the task of depth estimation under rainy and foggy conditions. All experiments are performed using the WeatherKITTI dataset, with both qualitative and quantitative analyses.

The results of the ablation study on the important components of the WeatherMono depth estimation network are presented in Table 4. Two groups of ablation experiments are designed, testing the model performance under rainy and foggy conditions, respectively.

From Table 4, we can observe that when the GLFI module is removed from stages 2, 3, and 4 of the encoder, the model’s complexity is reduced, but its depth estimation accuracy also decreases. This indicates that the GLFI module plays a crucial role in extracting global contextual information from images in rainy and foggy environments. The GLFI module effectively complements the CNN network’s limitation of only being able to extract local features, enabling the model to capture more comprehensive global information. Without it, the network’s ability to understand the broader context of the image is compromised, leading to less accurate depth estimations.

When the multi-scale deformable convolution (MDC) is replaced with a standard convolution module, the model’s depth estimation accuracy drops. This result further supports the importance of the MDC module in enhancing the extraction of local features and multi-scale information. The deformable convolution allows the network to adaptively focus on regions of interest and better capture the intricate details in rainy and foggy images. Additionally, by progressively extracting multi-scale features, MDC reduces the number of trainable parameters while improving the network’s ability to handle complex weather conditions.

Removing the three pooling connections results in a decrease in depth estimation accuracy. The pooling connection layers are crucial for retaining spatial feature information and preventing the loss of important spatial details when features are downsampled. Pooling helps the model preserve critical information throughout the network, ensuring that spatial details are not discarded, which is vital for accurate depth estimation in challenging environments like rain and fog.

The removal of the two cross-stage connections causes a slight decline in model accuracy. The purpose of these connections is to facilitate the propagation of features across different stages of the network, allowing for better feature fusion. The cross-stage connections improve feature utilization, enabling multi-stage features to be shared across different layers, which leads to improved depth estimation performance.

To further validate the effectiveness of the loss functions used in the rain and fog environment depth estimation task, we conduct three sets of ablation studies to analyze the impact of different loss functions on the model’s performance. The results, shown in Table 5, reveal that the inclusion of both image reconstruction loss and perceptual smoothness loss progressively enhances the depth estimation network’s overall performance.

Image Reconstruction Loss: Adding the image reconstruction loss improves the depth estimation accuracy, highlighting its role in guiding the model to recover spatial details of the image. The reconstruction loss helps refine the model’s ability to reconstruct the input image by minimizing the differences between the predicted and target images, which is crucial for improving the depth estimation, especially in the presence of rain and fog.

Perceptual Smoothness Loss: Adding the perceptual smoothness loss further improves model performance, indicating that it is effective in reducing noise and smoothing the transition areas of the depth map. This loss function helps mitigate inconsistencies in depth estimation caused by rain droplets and fog interference, ensuring smoother depth transitions and improving the robustness of the depth predictions.

From the ablation results, it is clear that both image reconstruction loss and perceptual smoothness loss play key roles in depth estimation under rainy and foggy conditions. The reconstruction loss aids in fine-tuning image detail restoration, while the perceptual smoothness loss alleviates the noise interference induced by weather conditions, significantly improving the robustness and accuracy of depth estimation in such environments.

## 5. Conclusions

This paper proposes a self-supervised learning network for monocular depth estimation in rainy and foggy environments, called WeatherMono. The network is based on a CNN-Transformer hybrid architecture aimed at improving the accuracy and robustness of monocular depth estimation in challenging weather conditions by combining the strengths of both network structures. Specifically, WeatherMono leverages the Transformer network to extract global context information from images while incorporating CNN networks to enhance multi-scale local features, effectively addressing the challenges of depth estimation in rainy and foggy environments. We have designed two novel modules: the Multi-Scale Deformable Convolution (MDC) module and the Global-Local Feature Interaction (GLFI) module. The MDC module adaptively adjusts the sampling locations of convolution kernels to effectively extract detailed local features in rainy and foggy environments, reducing the interference of occlusion and complex geometric structures on depth estimation. The GLFI module introduces an efficient multi-head attention mechanism in the Transformer encoder to enhance the expression of global features while reducing the computational complexity of traditional self-attention mechanisms, thus improving the performance of the monocular depth estimation network in rainy and foggy conditions.

Despite the promising results achieved by WeatherMono, several limitations should be acknowledged. First, the model is trained on synthetic data, and its generalization to diverse real-world weather conditions requires further validation. Second, the computational efficiency of the GLFI module, while improved, still exceeds that of pure CNN architectures, posing challenges for real-time deployment. Third, while our evaluation encompasses a range of weather intensities, a systematic breakdown of performance metrics by specific severity levels (e.g., light vs. heavy rain) would provide deeper insights into model behavior and failure modes. In terms of computational efficiency, exploring more lightweight attention mechanisms could further reduce complexity while maintaining performance. Regarding generalization, incorporating domain adaptation techniques or larger-scale real-world datasets would help address unseen weather variations. Extending WeatherMono to a multi-task learning framework that jointly handles depth estimation, object detection, and semantic segmentation under adverse weather is another valuable direction. Adapting the model to nighttime and low-light conditions, as well as compound effects such as rain at night, remains an important challenge. Finally, model compression techniques such as pruning and quantization could enable real-time deployment on resource-constrained platforms for autonomous driving applications.

## Figures and Tables

**Table 1:** Comparison of existing monocular depth estimation methods for adverse weather conditions.

Method	Architecture	Key Features	Limitations
Monodepth2	CNN	Multi-scale prediction, minimum reprojection loss	Poor global context capture
DepthFormer	Transformer	Multi-scale self-attention, global-local fusion	High computational complexity
MonoViT	CNN-Transformer	Hybrid architecture, position embeddings	Lacks specialized weather handling
Lite-Mono	CNN-Transformer	Lightweight design, efficient attention	Reduced capacity for complex weather
WeatherDepth	CNN	Curriculum contrastive learning	Limited local feature extraction
RoboDepth	CNN-Transformer	Robustness under corruptions	Not specialized for rain/fog
Md4all	CNN	Adversarial training for robustness	Limited global context
WeatherMono (Ours)	CNN-Transformer	MDC + GLFI modules, multi-scale deformable conv	(proposed method)

**Table 2:** Quantitative Comparison of Different Depth Estimation Algorithms on Test Datasets in Rainy and Foggy Environments.

Methods	Depth Error (↓)				Depth Accuracy (↑)		
	AbsRel	RMSE	RMSE log	SqRel	$a_{1}$	$a_{2}$	$a_{3}$
Monodepth2	0.168	8.171	0.229	2.123	0.768	0.922	0.972
DepthHints	0.164	8.065	0.227	2.117	0.774	0.926	0.976
DPT	0.161	7.918	0.218	1.902	0.776	0.931	0.977
FSRE-Depth	0.156	7.937	0.226	2.097	0.772	0.924	0.974
HR-Depth	0.163	7.929	0.224	1.917	0.771	0.926	0.975
ADDS-Depth	0.167	7.931	0.235	1.926	0.759	0.918	0.959
CADepth-Net	0.159	7.927	0.216	1.944	0.784	0.933	0.978
DiFFNet	0.146	7.884	0.200	1.668	0.805	0.945	0.983
Manydepth	0.161	7.929	0.221	1.912	0.761	0.921	0.967
MonoFormer	0.141	7.783	0.191	1.594	0.807	0.935	0.982
DepthFormer	0.138	7.594	0.181	1.468	0.811	0.933	0.981
DynaDepth	0.162	7.875	0.219	1.905	0.769	0.914	0.965
Lite-Mono	0.131	6.624	0.177	1.437	0.816	0.936	0.973
MonoVit	0.120	5.111	0.201	0.899	0.857	0.953	0.980
PlaneDepth	0.150	6.513	0.217	1.360	0.757	0.891	0.945
RoboDepth	0.107	4.604	0.183	0.791	0.883	0.963	0.983
Md4all	0.121	4.606	0.181	0.784	0.882	0.961	0.982
WeatherDepth	0.104	4.413	0.178	0.737	0.892	0.965	0.984
WeatherMono	0.097	4.281	0.162	0.702	0.897	0.976	0.989

**Table 3:** Quantitative Comparison with Adverse Environment Depth Estimation Algorithms in Real Rainy and Foggy Scenarios.

Methods	Depth Error (↓)				Depth Accuracy (↑)		
	AbsRel	RMSE	RMSE log	SqRel	$a_{1}$	$a_{2}$	$a_{3}$
(a) DrivingStereo Dataset:Rain							
ADDS-Depth	0.227	11.907	0.271	3.165	0.641	0.894	0.951
MonoVit	0.175	9.616	0.232	2.138	0.730	0.931	0.979
PlaneDepth	0.220	11.671	0.278	3.302	0.654	0.883	0.965
RoboDepth	0.167	9.157	0.221	2.019	0.755	0.938	0.982
Md4all	0.176	9.216	0.225	2.134	0.752	0.941	0.981
WeatherDepth	0.158	8.837	0.211	1.833	0.764	0.945	0.985
WeatherMono	0.149	8.634	0.202	1.824	0.775	0.951	0.988
(b) DrivingStereo Dataset:Fog							
ADDS-Depth	0.157	10.902	0.184	2.211	0.776	0.924	0.969
MonoVit	0.109	7.758	0.167	1.206	0.870	0.967	0.990
PlaneDepth	0.151	7.758	0.167	1.836	0.803	0.945	0.983
RoboDepth	0.105	7.276	0.158	1.135	0.882	0.974	0.992
Md4all	0.131	7.415	0.165	1.241	0.842	0.981	0.991
WeatherDepth	0.105	7.346	0.158	1.117	0.879	0.972	0.992
WeatherMono	0.101	7.265	0.144	1.112	0.881	0.988	0.992

**Table 4:** Quantitative Ablation Study of Each Component Module in the WeatherMono Depth Estimation Network Model.

Number	Architecture	Depth Error (↓)				Depth Accuracy (↑)		
		AbsRel	RMSE	RMSE log	SqRel	$a_{1}$	$a_{2}$	$a_{3}$
(a) DrivingStereo Dataset:Rain								
(a)	w/o GLFI	0.153	8.847	0.211	1.916	0.770	0.949	0.987
(b)	w/o MDC	0.155	8.742	0.212	1.906	0.769	0.949	0.987
(c)	w/o pooled connection	0.151	8.812	0.205	1.911	0.772	0.948	0.986
(d)	w/o cross-stage connections	0.150	8.753	0.204	1.914	0.773	0.949	0.987
(e)	WeatherMono(ours)	**0.149**	**8.634**	**0.202**	**1.824**	**0.775**	**0.951**	**0.988**
(b) DrivingStereo Dataset:Fog								
(a)	w/o GLFI	0.105	7.428	0.156	1.243	0.876	0.986	0.991
(b)	w/o MDC	0.108	7.314	0.158	1.201	0.875	0.986	0.990
(c)	w/o pooled connection	0.103	7.416	0.148	1.184	0.878	0.985	0.990
(d)	w/o cross-stage connections	0.102	7.354	0.146	1.211	0.879	0.986	0.991
(e)	WeatherMono(ours)	0.101	7.265	0.144	1.112	0.881	0.988	0.992

**Table 5:** Quantitative Ablation Study of the Loss Function in the WeatherMono Network Model.

Number	$L_{\mathrm{smooth}}$	$L_{r}$	Depth Error (↓)				Depth Accuracy (↑)		
			AbsRel	RMSE	RMSE log	SqRel	$a_{1}$	$a_{2}$	$a_{3}$
(a)	√		0.167	8.946	0.231	2.016	0.753	0.932	0.977
(b)		√	0.153	8.802	0.226	1.971	0.761	0.940	0.981
(c)	√	√	0.149	8.634	0.202	1.824	0.775	0.951	0.988

## Data Availability

Dataset available on request from the authors. The raw data supporting the conclusions of this article will be made available by the authors on request.

## References

[1] Zhang N., Nex F., Vosselman G., Kerle N. (2023). Lite-Mono: A Lightweight CNN and Transformer Architecture for Self-Supervised Monocular Depth Estimation. Proceedings of the 2023 IEEE/CVF Conference on Computer Vision and Pattern Recognition (CVPR), Vancouver, BC, Canada, 17–24 June 2023.

[2] Lahiri S., Ren J., Lin X. (2024). Deep learning-based stereopsis and monocular depth estimation techniques: A review. Vehicles.

[3] Li Z., Wang X., Liu X., Jiang J. (2022). Binsformer: Revisiting adaptive bins for monocular depth estimation. arXiv.

[4] Hu J., Cao Z. (2024). Monocular Depth Estimation Algorithm for Rainy Scenes. Proceedings of the 2024 5th International Conference on Electronic Communication and Artificial Intelligence (ICECAI), Guangzhou, China, 31 May–2 June 2024.

[5] Lee J., Lai-Dang Q.V., Sengar N., Har D. (2024). Robust Monocular Depth Estimation in Adverse Weather Conditions by Unsupervised Domain Adaptation. Proceedings of the ECAI 2024, Santiago de Compostela, Spain, 19–24 October 2024.

[6] Gasperini S., Morbitzer N., Jung H., Navab N., Tombari F. (2023). Robust monocular depth estimation under challenging conditions. Proceedings of the IEEE/CVF International Conference on Computer Vision, Paris, France, 2–6 October 2023.

[7] Liu J., Guo Z., Ping P., Zhang H., Shi Q. (2024). Channel Interaction and Transformer Depth Estimation Network: Robust Self-Supervised Depth Estimation Under Varied Weather Conditions. Sustainability.

[8] Saunders K., Vogiatzis G., Manso L.J. (2023). Self-supervised monocular depth estimation: Let’s talk about the weather. Proceedings of the IEEE/CVF International Conference on Computer Vision, Paris, France, 2–6 October 2023.

[9] Eigen D., Puhrsch C., Fergus R. (2014). Depth map prediction from a single image using a multi-scale deep network. arXiv.

[10] Laina I., Rupprecht C., Belagiannis V., Tombari F., Navab N. (2016). Deeper depth prediction with fully convolutional residual networks. Proceedings of the 2016 Fourth International Conference on 3D Vision (3DV), Stanford, CA, USA, 25–28 October 2016.

[11] Fu H., Gong M., Wang C., Batmanghelich K., Tao D. (2018). Deep ordinal regression network for monocular depth estimation. Proceedings of the IEEE Conference on Computer Vision and Pattern Recognition, Salt Lake City, UT, USA, 18–23 June 2018.

[12] Zhou T., Brown M., Snavely N., Lowe D.G. (2017). Unsupervised learning of depth and ego-motion from video. Proceedings of the IEEE Conference on Computer Vision and Pattern Recognition, Honolulu, HI, USA, 21–26 July 2017.

[13] Yin Z., Shi J. (2018). Geonet: Unsupervised learning of dense depth, optical flow and camera pose. Proceedings of the IEEE Conference on Computer Vision and Pattern Recognition, Salt Lake City, UT, USA, 18–23 June 2018.

[14] Jung H., Park E., Yoo S. (2021). Fine-grained semantics-aware representation enhancement for self-supervised monocular depth estimation. Proceedings of the IEEE/CVF International Conference on Computer Vision, Montreal, QC, Canada, 10–17 October 2021.

[15] Godard C., Mac Aodha O., Firman M., Brostow G.J. (2019). Digging into self-supervised monocular depth estimation. Proceedings of the IEEE/CVF International Conference on Computer Vision, Seoul, Republic of Korea, 27 October–2 November 2019.

[16] Zhou H., Greenwood D., Taylor S. (2021). Self-supervised monocular depth estimation with internal feature fusion. arXiv.

[17] Agarwal A., Arora C. (2022). Depthformer: Multiscale vision transformer for monocular depth estimation with global local information fusion. Proceedings of the 2022 IEEE International Conference on Image Processing (ICIP), Bordeaux, France, 16–19 October 2022.

[18] Ranftl R., Bochkovskiy A., Koltun V. (2021). Vision transformers for dense prediction. Proceedings of the IEEE/CVF International Conference on Computer Vision, Montreal, QC, Canada, 10–17 October 2021.

[19] Bae J., Moon S., Im S. (2022). Monoformer: Towards generalization of self-supervised monocular depth estimation with transformers. arXiv.

[20] Zhao C., Zhang Y., Poggi M., Tosi F., Guo X., Zhu Z., Huang G., Tang Y., Mattoccia S. (2022). Monovit: Self-supervised monocular depth estimation with a vision transformer. Proceedings of the 2022 International Conference on 3D Vision (3DV), Prague, Czechia, 12–15 September 2022.

[21] Liu L., Song X., Wang M., Liu Y., Zhang L. (2021). Self-supervised monocular depth estimation for all day images using domain separation. Proceedings of the CVF International Conference on Computer Vision (ICCV), Montreal, QC, Canada, 10–17 October 2021.

[22] Zhao C., Tang Y., Sun Q. (2022). Unsupervised monocular depth estimation in highly complex environments. IEEE Trans. Emerg. Top. Comput. Intell..

[23] Lee Y., Jeon J., Ko Y., Jeon B., Jeon M. (2021). Task-driven deep image enhancement network for autonomous driving in bad weather. Proceedings of the 2021 IEEE International Conference on Robotics and Automation (ICRA), Xi’an, China, 30 May–5 June 2021.

[24] Wang J., Lin C., Nie L., Huang S., Zhao Y., Pan X., Ai R. (2024). Weatherdepth: Curriculum contrastive learning for self-supervised depth estimation under adverse weather conditions. Proceedings of the 2024 IEEE International Conference on Robotics and Automation (ICRA), Yokohama, Japan, 13–17 May 2024.

[25] Zhou Z., Fan X., Shi P., Xin Y. (2021). R-msfm: Recurrent multi-scale feature modulation for monocular depth estimating. Proceedings of the IEEE/CVF International Conference on Computer Vision.

[26] Wang Z., Bovik A.C., Sheikh H.R., Simoncelli E.P. (2004). Image quality assessment: From error visibility to structural similarity. IEEE Trans. Image Process..

[27] Yang G., Song X., Huang C., Deng Z., Shi J., Zhou B. (2019). Drivingstereo: A large-scale dataset for stereo matching in autonomous driving scenarios. Proceedings of the IEEE/CVF Conference on Computer Vision and Pattern Recognition, Long Beach, CA, USA, 15–20 June 2019.

[28] Wang R., Yu Z., Gao S. (2023). Planedepth: Self-supervised depth estimation via orthogonal planes. Proceedings of the IEEE/CVF Conference on Computer Vision and Pattern Recognition, Vancouver, BC, Canada, 17–24 June 2023.

[29] Watson J., Firman M., Brostow G.J., Turmukhambetov D. (2019). Self-supervised monocular depth hints. Proceedings of the IEEE/CVF International Conference on Computer Vision, Seoul, Republic of Korea, 27 October–2 November 2019.

[30] Lyu X., Liu L., Wang M., Kong X., Liu L., Liu Y., Chen X., Yuan Y. (2021). Hr-depth: High resolution self-supervised monocular depth estimation. Proceedings of the AAAI Conference on Artificial Intelligence, Virtual, 19–21 May 2021.

[31] Yan J., Zhao H., Bu P., Jin Y. (2021). Channel-wise attention-based network for self-supervised monocular depth estimation. Proceedings of the 2021 International Conference on 3D Vision (3DV), Virtual, 1–3 December 2021.

[32] Watson J., Mac Aodha O., Prisacariu V., Brostow G., Firman M. (2021). The temporal opportunist: Self-supervised multi-frame monocular depth. Proceedings of the IEEE/CVF Conference on Computer Vision and Pattern Recognition, Nashville, TN, USA, 20–25 June 2021.

[33] Zhang S., Zhang J., Tao D. (2022). Towards scale-aware, robust, and generalizable unsupervised monocular depth estimation by integrating IMU motion dynamics. Proceedings of the European Conference on Computer Vision, Tel Aviv, Israel, 23–27 October 2022.

[34] Kong L., Xie S., Hu H., Ng L.X., Cottereau B., Ooi W.T. (2023). Robodepth: Robust out-of-distribution depth estimation under corruptions. Adv. Neural Inf. Process. Syst..

